# Examining the role of community resilience and social capital on mental health in public health emergency and disaster response: a scoping review

**DOI:** 10.1186/s12889-023-17242-x

**Published:** 2023-12-12

**Authors:** C. E. Hall, H. Wehling, J. Stansfield, J. South, S. K. Brooks, N. Greenberg, R. Amlôt, D. Weston

**Affiliations:** 1 Behavioural Science and Insights Unit, Evaluation & Translation Directorate, Science Group, UK Health Security Agency, Porton Down, Salisbury, SP4 0JG UK; 3 Health Protection Research Unit, Institute of Psychology, Psychiatry and Neuroscience, King’s College London, 10 Cutcombe Road, London, SE5 9RJ UK; 2 School of Health and Community Studies, Leeds Beckett University, Portland Building, PD519, Portland Place, Leeds, LS1 3HE UK; 4 King’s Centre for Military Health Research, Institute of Psychology, Psychiatry and Neuroscience, King’s College London, 10 Cutcombe Road, London, SE5 9RJ UK

**Keywords:** Community resilience, Social capital, Mental health, Community cohesion, Public health emergency, *Disaster*, COVID-19, Recovery, Recommendations

## Abstract

The ability of the public to remain psychologically resilient in the face of public health emergencies and disasters (such as the COVID-19 pandemic) is a key factor in the effectiveness of a national response to such events. Community resilience and social capital are often perceived as beneficial and ensuring that a community is socially and psychologically resilient may aid emergency response and recovery. This review presents a synthesis of literature which answers the following research questions: How are community resilience and social capital quantified in research?; What is the impact of community resilience on mental wellbeing?; What is the impact of infectious disease outbreaks, disasters and emergencies on community resilience and social capital?; and, What types of interventions enhance community resilience and social capital?

A scoping review procedure was followed. Searches were run across Medline, PsycInfo, and EMBASE, with search terms covering both community resilience and social capital, public health emergencies, and mental health. 26 papers met the inclusion criteria.

The majority of retained papers originated in the USA, used a survey methodology to collect data, and involved a natural disaster. There was no common method for measuring community resilience or social capital. The association between community resilience and social capital with mental health was regarded as positive in most cases. However, we found that community resilience, and social capital, were initially negatively impacted by public health emergencies and enhanced by social group activities.

Several key recommendations are proposed based on the outcomes from the review, which include: the need for a standardised and validated approach to measuring both community resilience and social capital; that there should be enhanced effort to improve preparedness to public health emergencies in communities by gauging current levels of community resilience and social capital; that community resilience and social capital should be bolstered if areas are at risk of disasters or public health emergencies; the need to ensure that suitable short-term support is provided to communities with high resilience in the immediate aftermath of a public health emergency or disaster; the importance of conducting robust evaluation of community resilience initiatives deployed during the COVID-19 pandemic.

**Supplementary Information:**

The online version contains supplementary material available at 10.1186/s12889-023-17242-x.

## Background

For the general population, public health emergencies and disasters (e.g., natural disasters; infectious disease outbreaks; Chemical, Biological, Radiological or Nuclear incidents) can give rise to a plethora of negative outcomes relating to both health (e.g. increased mental health problems [1–4]) and the economy (e.g., increased unemployment and decreased levels of tourism [4–6]). COVID-19 is a current, and ongoing, example of a public health emergency which has affected over 421 million individuals worldwide [7]. The long term implications of COVID-19 are not yet known, but there are likely to be repercussions for physical health, mental health, and other non-health related outcomes for a substantial time to come [8, 9]. As a result, it is critical to establish methods which may inform approaches to alleviate the longer-term negative consequences that are likely to emerge in the aftermath of both COVID-19 and any future public health emergency.

The definition of resilience often differs within the literature, but ultimately resilience is considered a dynamic process of adaptation. It is related to processes and capabilities at the individual, community and system level that result in good health and social outcomes, in spite of negative events, serious threats and hazards [10]. Furthermore, Ziglio [10] refers to four key types of resilience capacity: adaptive, the ability to withstand and adjust to unfavourable conditions and shocks; absorptive, the ability to withstand but also to recover and manage using available assets and skills; anticipatory, the ability to predict and minimize vulnerability; and transformative, transformative change so that systems better cope with new conditions.

There is no one settled definition of community resilience (CR). However, it generally relates to the ability of a community to withstand, adapt and permit growth in adverse circumstances due to social structures, networks and interdependencies within the community [11]. Social capital (SC) is considered a major determinant of CR [12, 13], and reflects strength of a social network, community reciprocity, and trust in people and institutions [14]. These aspects of community are usually conceptualised primarily as protective factors that enable communities to cope and adapt collectively to threats. SC is often broken down into further categories [15], for example: cognitive SC (i.e. perceptions of community relations, such as trust, mutual help and attachment) and structural SC (i.e. what actually happens within the community, such as participation, socialising) [16]; or, bonding SC (i.e. connections among individuals who are emotionally close, and result in bonds to a particular group [17]) and bridging SC (i.e. acquaintances or individuals loosely connected that span different social groups [18]). Generally, CR is perceived to be primarily beneficial for multiple reasons (e.g. increased social support [18, 19], protection of mental health [20, 21]), and strengthening community resilience is a stated health goal of the World Health Organisation [22] when aiming to alleviate health inequalities and protect wellbeing. This is also reflected by organisations such as Public Health England (now split into the UK Health Security Agency and the Office for Health Improvement and Disparities) [23] and more recently, CR has been targeted through the endorsement of Community Champions (who are volunteers trained to support and to help improve health and wellbeing. Community Champions also reflect their local communities in terms of population demographics for example age, ethnicity and gender) as part of the COVID-19 response in the UK (e.g. [24, 25]).

Despite the vested interest in bolstering communities, the research base establishing: how to understand and measure CR and SC; the effect of CR and SC, both during and following a public health emergency (such as the COVID-19 pandemic); and which types of CR or SC are the most effective to engage, is relatively small. Given the importance of ensuring resilience against, and swift recovery from, public health emergencies, it is critically important to establish and understand the evidence base for these approaches. As a result, the current review sought to answer the following research questions: (1) How are CR and SC quantified in research?; (2) What is the impact of community resilience on mental wellbeing?; (3) What is the impact of infectious disease outbreaks, disasters and emergencies on community resilience and social capital?; and, (4) What types of interventions enhance community resilience and social capital?

By collating research in order to answer these research questions, the authors have been able to propose several key recommendations that could be used to both enhance and evaluate CR and SC effectively to facilitate the long-term recovery from COVID-19, and also to inform the use of CR and SC in any future public health disasters and emergencies.

## Method

A scoping review methodology was followed due to the ease of summarising literature on a given topic for policy makers and practitioners [26], and is detailed in the following sections.

### Identification of relevant studies

An initial search strategy was developed by authors CH and DW and included terms which related to: CR and SC, given the absence of a consistent definition of CR, and the link between CR and SC, the review focuses on both CR and SC to identify as much relevant literature as possible (adapted for purpose from Annex 1: [27], as well as through consultation with review commissioners); public health emergencies and disasters [28–31], and psychological wellbeing and recovery (derived a priori from literature). To ensure a focus on both public health and psychological research, the final search was carried across Medline, PsycInfo, and EMBASE using OVID. The final search took place on the 18th of May 2020, the search strategy used for all three databases can be found in Supplementary file 1.

### Selection criteria

The inclusion and exclusion criteria were developed alongside the search strategy. Initially the criteria were relatively inclusive and were subject to iterative development to reflect the authors’ familiarisation with the literature. For example, the decision was taken to exclude research which focused exclusively on social support and did not mention communities as an initial title/abstract search suggested that the majority of this literature did not meet the requirements of our research question.

The full and final inclusion and exclusion criteria used can be found in Supplementary file 2. In summary, authors decided to focus on the general population (i.e., non-specialist, e.g. non-healthcare worker or government official) to allow the review to remain community focused. The research must also have assessed the impact of CR and/or SC on mental health and wellbeing, resilience, and recovery during and following public health emergencies and infectious disease outbreaks which affect communities (to ensure the research is relevant to the review aims), have conducted primary research, and have a full text available or provided by the first author when contacted.

### Charting the data

All papers were first title and abstract screened by CH or DW. Papers then were full text reviewed by CH to ensure each paper met the required eligibility criteria, if unsure about a paper it was also full text reviewed by DW. All papers that were retained post full-text review were subjected to a standardised data extraction procedure. A table was made for the purpose of extracting the following data: title, authors, origin, year of publication, study design, aim, disaster type, sample size and characteristics, variables examined, results, restrictions/limitations, and recommendations. Supplementary file 3 details the charting the data process.

### Analytical method

Data was synthesised using a Framework approach [32], a common method for analysing qualitative research. This method was chosen as it was originally used for large-scale social policy research [33] as it seeks to identify: what works, for whom, in what conditions, and why [34]. This approach is also useful for identifying commonalities and differences in qualitative data and potential relationships between different parts of the data [33]. An a priori framework was established by CH and DW. Extracted data was synthesised in relation to each research question, and the process was iterative to ensure maximum saturation using the available data.

## Results

### Study selection

**Fig. 1:**
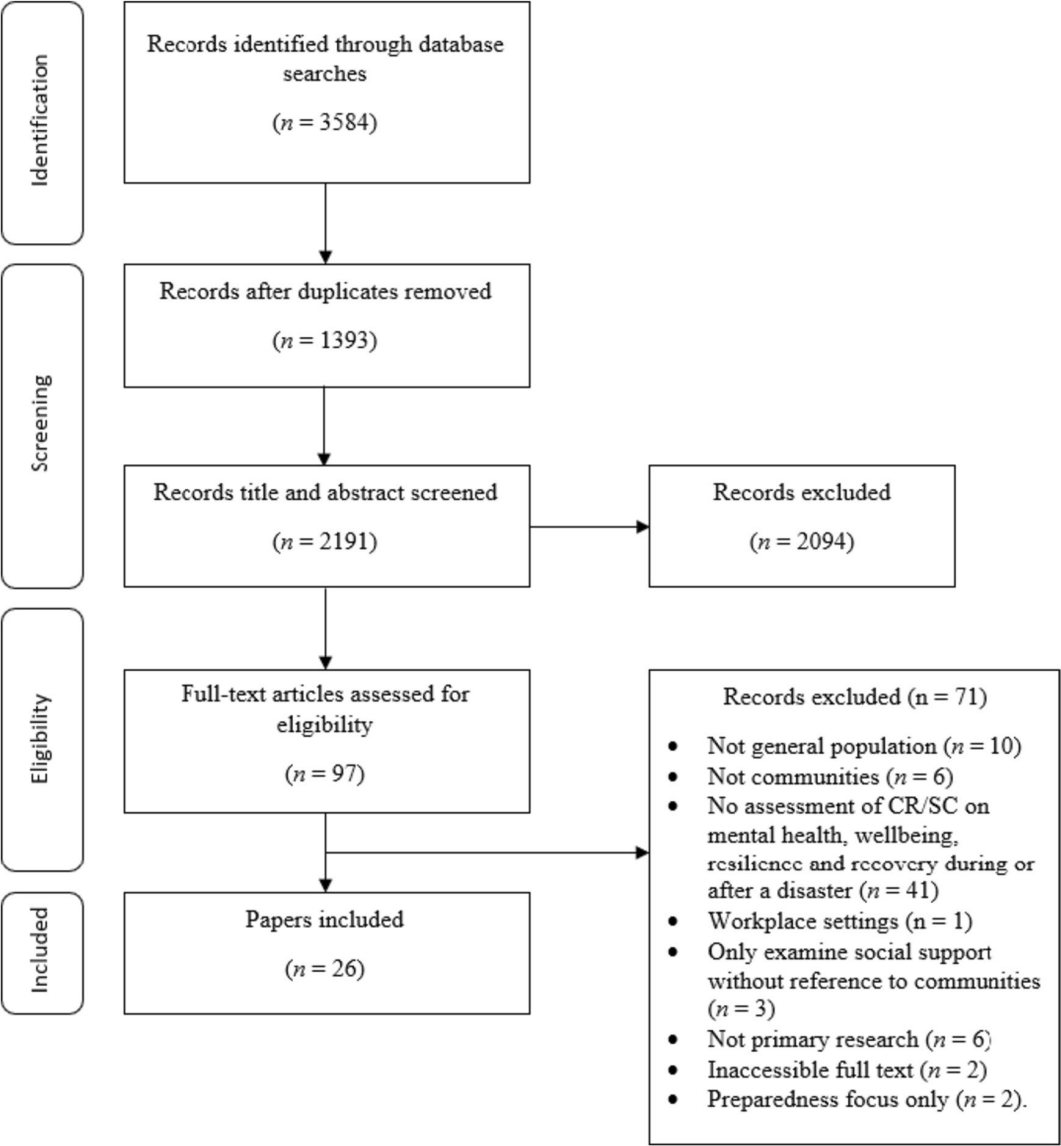
PRISMA flow diagram

The final search strategy yielded 3584 records. Following the removal of duplicates, 2191 records remained and were included in title and abstract screening. A PRISMA flow diagram is presented in Fig. 1.

At the title and abstract screening stage, the process became more iterative as the inclusion criteria were developed and refined. For the first iteration of screening, CH or DW sorted all records into ‘include,’ ‘exclude,’ and ‘unsure’. All ‘unsure’ papers were re-assessed by CH, and a random selection of ~ 20% of these were also assessed by DW. Where there was disagreement between authors the records were retained, and full text screened. The remaining papers were reviewed by CH, and all records were categorised into ‘include’ and ‘exclude’. Following full-text screening, 26 papers were retained for use in the review.

### Study characteristics

This section of the review addresses study characteristics of those which met the inclusion criteria, which comprises: date of publication, country of origin, study design, study location, disaster, and variables examined.

#### Date of publication

**Fig. 2:**
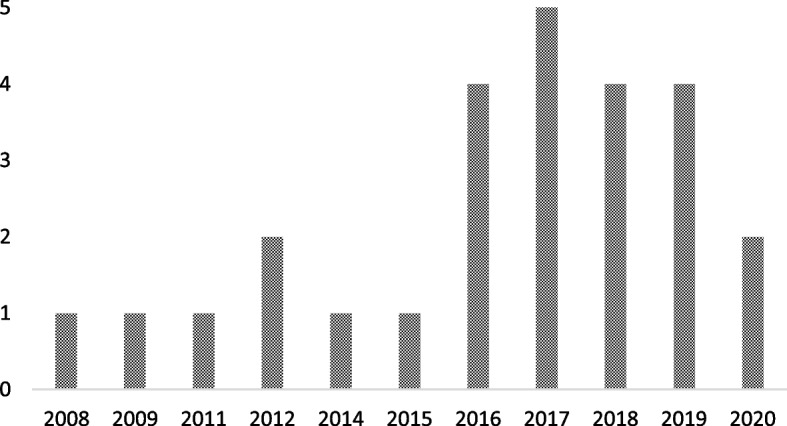
Graph to show retained papers date of publication

Publication dates across the 26 papers spanned from 2008 to 2020 (see Fig. 2). The number of papers published was relatively low and consistent across this timescale (i.e. 1–2 per year, except 2010 and 2013 when none were published) up until 2017 where the number of papers peaked at 5. From 2017 to 2020 there were 15 papers published in total. The amount of papers published in recent years suggests a shift in research and interest towards CR and SC in a disaster/ public health emergency context.

#### Country of origin

The locations of the first authors’ institutes at the time of publication were extracted to provide a geographical spread of the retained papers. The majority originated from the USA [35–41], followed by China [42–46], Japan [47–50], Australia [51–53], The Netherlands [54, 55], New Zealand [56], Peru [57], Iran [58], Austria [59], and Croatia [60].

#### Design

There were multiple methodological approaches carried out across retained papers. The most common formats included surveys or questionnaires [36–38, 42, 46–50, 53–55, 57, 59], followed by interviews [39, 40, 43, 51, 52, 60]. Four papers used both surveys and interviews [35, 41, 45, 58], and two papers conducted data analysis (one using open access data from a Social Survey [44] and one using a Primary Health Organisations Register [56]).

#### Study location

The majority of the studies were carried out in Japan [36, 42, 44, 47–50], followed by the USA [35, 37–41], China [43, 45, 46, 53], Australia [51, 52], and the UK [54, 55]. The remaining studies were carried out in Croatia [60], Peru [57], Austria [59], New Zealand [56] and Iran [58].

#### Disaster

Multiple different types of disaster were researched across the retained papers. Earthquakes were the most common type of disaster examined [45, 47, 49, 50, 53, 56–58], followed by research which assessed the impact of two disastrous events which had happened in the same area (e.g. Hurricane Katrina and the Deepwater Horizon oil spill in Mississippi, and the Great East Japan earthquake and Tsunami; [36–38, 42, 44, 48]). Other disaster types included: flooding [51, 54, 55, 59, 60], hurricanes [35, 39, 41], infectious disease outbreaks [43, 46], oil spillage [40], and drought [52].

#### Variables of interest examined

Across the 26 retained papers: eight referred to examining the impact of SC [35, 37, 39, 41, 46, 49, 55, 60]; eight examined the impact of cognitive and structural SC as separate entities [40, 42, 45, 48, 50, 54, 57, 59]; one examined bridging and bonding SC as separate entities [58]; two examined the impact of CR [38, 56]; and two employed a qualitative methodology but drew findings in relation to bonding and bridging SC, and SC generally [51, 52]. Additionally, five papers examined the impact of the following variables: ‘community social cohesion’ [36], ‘neighbourhood connectedness’ [44], ‘social support at the community level’ [47], ‘community connectedness’ [43] and ‘sense of community’ [53]. Table 1 provides additional details on this.

**Table 1:** Measures used by retained papers to examine CR and SC.

Variable	Measure	Reference
SC	Social capital and Community Engagement subscale of the Community Resources Scale	[60]
	American Community Survey five-year estimate	[39]
	12-item instrument that includes perceived availability of different types of assistance during disaster(s)	[37]
	The PSCI-16	[46]
	Six scales adapted scales from another research study.	[41]
	SA-SCAT	[55]
	Community-level social capital was obtained as the average score of individual responses to a social capital scale	[49]
	Neighbourhood Collective Efficacy Scale	[35]
Structural and Cognitive SC	SA-SCAT	[54, 57]
	Cognitive social capital measure involved measures of perceived trust, fairness, helpfulness, consideration, participation and community involvement. Structural social capital was measured using estimates on provided and received help measured in person-days during the response and recovery phase.	[59]
	Questionnaire with items measuring: residents’ perceptions of trust in the community, norms of mutual help, and community attachment; and the frequency of meeting with friends, the number of friends with whom the respondent met during the past month, and the frequency participating in sports and hobby clubs per week	[42]
	Items derived from the Sense of Community Index and Informal Social Control. Community participation.	[40]
	Measure of generalised trust: i.e. Would you say that most people can generally be trusted?	[50]
	Structural social capital was measured using the Association Scale, which was adapted from the Social Network and Association Scales. Cognitive social capital was measured according to respondents’ perception of three aspects, namely sense of community, trust, and social connectedness.	[45]
	Cognitive social capital was measured by asking about perception of fairness, trust and reciprocity toward the community a participant belonged to. Structural social capital was measured by social support and social participation.	[48]
Bonding and Bridging SC	Persian version of the Bonding and Bridging social capital Questionnaire	[58]
CR	Communities Advancing Resilience Toolkit (CART)	[38]
	Community resilience mapping and earthquake impact analysis sections of the 2011 and 2012 community profiles created by the Christchurch Community Advisors	[56]
Community Social Cohesion	Questionnaire relating to residents’ perceptions of trust in the community, levels of mutual help, and community attachment	[36]
Neighbourhood Connectedness	Two questions asked: How often do you talk with your neighbours? How often do you tell your neighbours about your troubles or vice versa?	[44]
Social Support at the Community level	Calculation relating to social support and social participation by prefabricated temporary housing communities	[47]
Community Connectedness	Personal Wellbeing Index	[43]
Sense of Community	Brief Sense of Community Scale	[53]

### How is CR and SC measured or quantified in research?

The measures used to examine CR and SC are presented Table 1. It is apparent that there is no uniformity in how SC or CR is measured across the research. Multiple measures are used throughout the retained studies, and nearly all are unique. Additionally, SC was examined at multiple different levels (e.g. cognitive and structural, bonding and bridging), and in multiple different forms (e.g. community connectedness, community cohesion).

### What is the association between CR and SC on mental wellbeing?

To best compare research, the following section reports on CR, and facets of SC separately. Please see Supplementary file 4 for additional information on retained papers methods of measuring mental wellbeing.

#### Community resilience

CR relates to the ability of a community to withstand, adapt and permit growth in adverse circumstances due to social structures, networks and interdependencies within the community [11].

The impact of CR on mental wellbeing was consistently positive. For example, research indicated that there was a positive association between CR and number of common mental health (i.e. anxiety and mood) treatments post-disaster [56]. Similarly, other research suggests that CR is positively related to psychological resilience, which is inversely related to depressive symptoms) [37]. The same research also concluded that CR is protective of psychological resilience and is therefore protective of depressive symptoms [37].

#### Social capital

SC reflects the strength of a social network, community reciprocity, and trust in people and institutions [14]. These aspects of community are usually conceptualised primarily as protective factors that enable communities to cope and adapt collectively to threats.

There were inconsistencies across research which examined the impact of abstract SC (i.e. not refined into bonding/bridging or structural/cognitive) on mental wellbeing. However, for the majority of cases, research deems SC to be beneficial. For example, research has concluded that, SC is protective against post-traumatic stress disorder [55], anxiety [46], psychological distress [50], and stress [46]. Additionally, SC has been found to facilitate post-traumatic growth [38], and also to be useful to be drawn upon in times of stress [52], both of which could be protective of mental health. Similarly, research has also found that emotional recovery following a disaster is more difficult for those who report to have low levels of SC [51].

Conversely, however, research has also concluded that when other situational factors (e.g. personal resources) were controlled for, a positive relationship between community resources and life satisfaction was no longer significant [60]. Furthermore, some research has concluded that a high level of SC can result in a community facing greater stress immediately post disaster. Indeed, one retained paper found that high levels of SC correlate with higher levels of post-traumatic stress immediately following a disaster [39]. However, in the later stages following a disaster, this relationship can reverse, with SC subsequently providing an aid to recovery [41]. By way of explanation, some researchers have suggested that communities with stronger SC carry the greatest load in terms of helping others (i.e. family, friends and neighbours) as well as themselves immediately following the disaster, but then as time passes the communities recover at a faster rate as they are able to rely on their social networks for support [41].

#### Cognitive and structural social capital

Cognitive SC refers to perceptions of community relations, such as trust, mutual help and attachment, and structural SC refers to what actually happens within the community, such as participation, socialising [16].

Cognitive SC has been found to be protective [49] against PTSD [54, 57], depression [40, 54]) mild mood disorder; [48]), anxiety [48, 54] and increase self-efficacy [59].

For structural SC, research is again inconsistent. On the one hand, structural SC has been found to: increase perceived self-efficacy, be protective of depression [40], buffer the impact of housing damage on cognitive decline [42] and provide support during disasters and over the recovery period [59]. However, on the other hand, it has been found to have no association with PTSD [54, 57] or depression, and is also associated with a higher prevalence of anxiety [54]. Similarly, it is also suggested by additional research that structural SC can harm women’s mental health, either due to the pressure of expectations to help and support others or feelings of isolation [49].

#### Bonding and bridging social capital

Bonding SC refers to connections among individuals who are emotionally close, and result in bonds to a particular group [17], and bridging SC refers to acquaintances or individuals loosely connected that span different social groups [18].

One research study concluded that both bonding and bridging SC were protective against post-traumatic stress disorder symptoms [58]. Bridging capital was deemed to be around twice as effective in buffering against post-traumatic stress disorder than bonding SC [58].

#### Other community variables

Community social cohesion was significantly associated with a lower risk of post-traumatic stress disorder symptom development [35], and this was apparent even whilst controlling for depressive symptoms at baseline and disaster impact variables (e.g. loss of family member or housing damage) [36]. Similarly, sense of community, community connectedness, social support at the community level and neighbourhood connectedness all provided protective benefits for a range of mental health, wellbeing and recovery variables, including: depression [53], subjective wellbeing (in older adults only) [43], psychological distress [47], happiness [44] and life satisfaction [53].

Research has also concluded that community level social support is protective against mild mood and anxiety disorder, but only for individuals who have had no previous disaster experience [48]. Additionally, a study which separated SC into social cohesion and social participation concluded that at a community level, social cohesion is protective against depression [49] whereas social participation at community level is associated with an increased risk of depression amongst women [49].

### What is the impact of Infectious disease outbreaks / disasters and emergencies on community resilience?

From a cross-sectional perspective, research has indicated that disasters and emergencies can have a negative effect on certain types of SC. Specifically, cognitive SC has been found to be impacted by disaster impact, whereas structural SC has gone unaffected [45]. Disaster impact has also been shown to have a negative effect on community relationships more generally [52].

Additionally, of the eight studies which collected data at multiple time points [35, 36, 41, 42, 47, 49, 56, 60], three reported the effect of a disaster on the level of SC within a community [40, 42, 49]. All three of these studies concluded that disasters may have a negative impact on the levels of SC within a community. The first study found that the Deepwater Horizon oil spill had a negative effect on SC and social support, and this in turn explained an overall increase in the levels of depression within the community [40]. A possible explanation for the negative effect lays in ‘corrosive communities’, known for increased social conflict and reduced social support, that are sometimes created following oil spills [40]. It is proposed that corrosive communities often emerge due to a loss of natural resources that bring social groups together (e.g., for recreational activities), as well as social disparity (e.g., due to unequal distribution of economic impact) becoming apparent in the community following disaster [40]. The second study found that SC (in the form of social cohesion, informal socialising and social participation) decreased after the 2011 earthquake and tsunami in Japan; it was suggested that this change correlated with incidence of cognitive decline [42]. However, the third study reported more mixed effects based on physical circumstances of the communities’ natural environment: Following an earthquake, those who lived in mountainous areas with an initial high level of pre-community SC saw a decrease in SC post disaster [49]. However, communities in flat areas (which were home to younger residents and had a higher population density) saw an increase in SC [49]. It was proposed that this difference could be due to the need for those who lived in mountainous areas to seek prolonged refuge due to subsequent landslides [49].

### What types of intervention enhance CR and SC and protect survivors?

There were mixed effects across the 26 retained papers when examining the effect of CR and SC on mental wellbeing. However, there is evidence that an increase in SC [56, 57], with a focus on cognitive SC [57], namely by: building social networks [45, 51, 53], enhancing feelings of social cohesion [35, 36] and promoting a sense of community [53], can result in an increase in CR and potentially protect survivors’ wellbeing and mental health following a disaster. An increase in SC may also aid in decreasing the need for individual psychological interventions in the aftermath of a disaster [55]. As a result, recommendations and suggested methods to bolster CR and SC from the retained papers have been extracted and separated into general methods, preparedness and policy level implementation.

#### General methods

Suggested methods to build SC included organising recreational activity-based groups [44] to broaden [51, 53] and preserve current social networks [42], introducing initiatives to increase social cohesion and trust [51], and volunteering to increase the number of social ties between residents [59]. Research also notes that it is important to take a ‘no one left behind approach’ when organising recreational and social community events, as failure to do so could induce feelings of isolation for some members of the community [49]. Furthermore, gender differences should also be considered as research indicates that males and females may react differently to community level SC (as evidence suggests males are instead more impacted by individual level SC; in comparison to women who have larger and more diverse social networks [49]). Therefore, interventions which aim to raise community level social participation, with the aim of expanding social connections and gaining support, may be beneficial [42, 47].

#### Preparedness

In order to prepare for disasters, it may be beneficial to introduce community-targeted methods or interventions to increase levels of SC and CR as these may aid in ameliorating the consequences of a public health emergency or disaster [57]. To indicate which communities have low levels of SC, one study suggests implementing a 3-item scale of social cohesion to map areas and target interventions [42].

It is important to consider that communities with a high level of SC may have a lower level of risk perception, due to the established connections and supportive network they have with those around them [61]. However, for the purpose of preparedness, this is not ideal as perception of risk is a key factor when seeking to encourage behavioural adherence. This could be overcome by introducing communication strategies which emphasise the necessity of social support, but also highlights the need for additional measures to reduce residual risk [59]. Furthermore, support in the form of financial assistance to foster current community initiatives may prove beneficial to rural areas, for example through the use of an asset-based community development framework [52].

#### Policy level

At a policy level, the included papers suggest a range of ways that CR and SC could be bolstered and used. These include: providing financial support for community initiatives and collective coping strategies, (e.g. using asset-based community development [52]); ensuring policies for long-term recovery focus on community sustainable development (e.g. community festival and community centre activities) [44]; and development of a network amongst cooperative corporations formed for reconstruction and to organise self-help recovery sessions among residents of adjacent areas [58].

## Discussion

This scoping review sought to synthesise literature concerning the role of SC and CR during public health emergencies and disasters. Specifically, in this review we have examined: the methods used to measure CR and SC; the impact of CR and SC on mental wellbeing during disasters and emergencies; the impact of disasters and emergencies on CR and SC; and the types of interventions which can be used to enhance CR. To do this, data was extracted from 26 peer-reviewed journal articles. From this synthesis, several key themes have been identified, which can be used to develop guidelines and recommendations for deploying CR and SC in a public health emergency or disaster context. These key themes and resulting recommendations are summarised below.

Firstly, this review established that there is no consistent or standardised approach to measuring CR or SC within the general population. This finding is consistent with a review conducted by the World Health Organization which concludes that despite there being a number of frameworks that contain indicators across different determinants of health, there is a lack of consensus on priority areas for measurement and no widely accepted indicator [27]. As a result, there are many measures of CR and SC apparent within the literature (e.g., [62, 63]), an example of a developed and validated measure is provided by Sherrieb, Norris and Galea [64]. Similarly, the definitions of CR and SC differ widely between researchers, which created a barrier to comparing and summarising information. Therefore, future research could seek to compare various interpretations of CR and to identify any overlapping concepts. However, a previous systemic review conducted by Patel et al. (2017) concludes that there are nine core elements of CR (local knowledge, community networks and relationships, communication, health, governance and leadership, resources, economic investment, preparedness, and mental outlook), with 19 further sub-elements therein [30]. Therefore, as CR is a multi-dimensional construct, the implications from the findings are that multiple aspects of social infrastructure may need to be considered.

Secondly, our synthesis of research concerning the role of CR and SC for ensuring mental health and wellbeing during, or following, a public health emergency or disaster revealed mixed effects. Much of the research indicates either a generally protective effect on mental health and wellbeing, or no effect; however, the literature demonstrates some potential for a high level of CR/SC to backfire and result in a negative effect for populations during, or following, a public health emergency or disaster. Considered together, our synthesis indicates that cognitive SC is the only facet of SC which was perceived as universally protective across all retained papers. This is consistent with a systematic review which also concludes that: (a) community level cognitive SC is associated with a lower risk of common mental disorders, while; (b) community level structural SC had inconsistent effects [65].

Further examination of additional data extracted from studies which found that CR/SC had a negative effect on mental health and wellbeing revealed no commonalities that might explain these effects (Please see Supplementary file 5 for additional information)

One potential explanation may come from a retained paper which found that high levels of SC result in an increase in stress level immediately post disaster [41]. This was suggested to be due to individuals having greater burdens due to wishing to help and support their wide networks as well as themselves. However, as time passes the levels of SC allow the community to come together and recover at a faster rate [41]. As this was the only retained paper which produced this finding, it would be beneficial for future research to examine boundary conditions for the positive effects of CR/SC; that is, to explore circumstances under which CR/SC may be more likely to put communities at greater risk. This further research should also include additional longitudinal research to validate the conclusions drawn by [41] as resilience is a dynamic process of adaption.

Thirdly, disasters and emergencies were generally found to have a negative effect on levels of SC. One retained paper found a mixed effect of SC in relation to an earthquake, however this paper separated participants by area in which they lived (i.e., mountainous vs. flat), which explains this inconsistent effect [49]. Dangerous areas (i.e. mountainous) saw a decrease in community SC in comparison to safer areas following the earthquake (an effect the authors attributed to the need to seek prolonged refuge), whereas participants from the safer areas (which are home to younger residents with a higher population density) saw an increase in SC [49]. This is consistent with the idea that being able to participate socially is a key element of SC [12]. Overall, however, this was the only retained paper which produced a variable finding in relation to the effect of disaster on levels of CR/SC.

Finally, research identified through our synthesis promotes the idea of bolstering SC (particularly cognitive SC) and cohesion in communities likely to be affected by disaster to improve levels of CR. This finding provides further understanding of the relationship between CR and SC; an association that has been reported in various articles seeking to provide conceptual frameworks (e.g., [66, 67]) as well as indicator/measurement frameworks [27]. Therefore, this could be done by creating and promoting initiatives which foster SC and create bonds within the community. Papers included in the current review suggest that recreational-based activity groups and volunteering are potential methods for fostering SC and creating community bonds [44, 51, 59]. Similarly, further research demonstrates that feelings of social cohesion are enhanced by general social activities (e.g. fairs and parades [18]). Also, actively encouraging activities, programs and interventions which enhance connectedness and SC have been reported to be desirable to increase CR [68]. This suggestion is supported by a recent scoping review of literature [67] examined community champion approaches for the COVID-19 pandemic response and recovery and established that creating and promoting SC focused initiatives within the community during pandemic response is highly beneficial [67]. In terms of preparedness, research states that it may be beneficial for levels of SC and CR in communities at risk to be assessed, to allow targeted interventions where the population may be at most risk following an incident [42, 44]. Additionally, from a more critical perspective, we acknowledge that ‘resilience’ can often be perceived as a focus on individual capacity to adapt to adversity rather than changing or mitigating the causes of adverse conditions [69, 70]. Therefore, CR requires an integrated system approach across individual, community and structural levels [17]. Also, it is important that community members are engaged in defining and agreeing how community resilience is measured [27] rather than it being imposed by system leads or decision-makers.

In the aftermath of the pandemic, is it expected that there will be long-term repercussions both from an economic [8] and a mental health perspective [71]. Furthermore, the findings from this review suggest that although those in areas with high levels of SC may be negatively affected in the acute stage, as time passes, they have potential to rebound at a faster rate than those with lower levels of SC. Ongoing evaluation of the effectiveness of current initiatives as the COVID-19 pandemic progresses into a recovery phase will be invaluable for supplementing the evidence base identified through this review.

## Recommendations

As a result of this review, a number of recommendations are suggested for policy and practice during public health emergencies and recovery.

- Future research should seek to establish a standardised and validated approach to measuring and defining CR and SC within communities. There are ongoing efforts in this area, for example [72]. Additionally, community members should be involved in the process of defining how CR is measured.
- There should be an enhanced effort to improve preparedness for public health emergencies and disasters in local communities by gauging current levels of SC and CR within communities using a standardised measure. This approach could support specific targeting of populations with low levels of CR/SC in case of a disaster or public health emergency, whilst also allowing for consideration of support for those with high levels of CR (as these populations can be heavily impacted initially following a disaster). By distinguishing levels of SC and CR, tailored community-centred approaches could be implemented, such as those listed in a guide released by PHE in 2015 [73].
- CR and SC (specifically cognitive SC) should be bolstered if communities are at risk of experiencing a disaster or public health emergency. This can be achieved by using interventions which aim to increase a sense of community and create new social ties (e.g., recreational group activities, volunteering). Additionally, when aiming to achieve this, it is important to be mindful of the risk of increased levels of CR/SC to backfire, as well as seeking to advocate an integrated system approach across individual, community and structural levels.
- It is necessary to be aware that although communities with high existing levels of resilience / SC may experience short-term negative consequences following a disaster, over time these communities might be able to recover at a faster rate. It is therefore important to ensure that suitable short-term support is provided to these communities in the immediate aftermath of a public health emergency or disaster.
- Robust evaluation of the community resilience initiatives deployed during the COVID-19 pandemic response is essential to inform the evidence base concerning the effectiveness of CR/ SC. These evaluations should continue through the response phase and into the recovery phase to help develop our understanding of the long-term consequences of such interventions.

### Limitations

Despite this review being the first in this specific topic area, there are limitations that must be considered. Firstly, it is necessary to note that communities are generally highly diverse and the term ‘community’ in academic literature is a subject of much debate (see: [74]), therefore this must be considered when comparing and collating research involving communities. Additionally, the measures of CR and SC differ substantially across research, including across the 26 retained papers used in the current review. This makes the act of comparing and collating research findings very difficult. This issue is highlighted as a key outcome from this review, and suggestions for how to overcome this in future research are provided. Additionally, we acknowledge that there will be a relationship between CR & SC even where studies measure only at individual or community level. A review [75] on articulating a hypothesis of the link to health inequalities suggests that wider structural determinants of health need to be accounted for. Secondly, despite the final search strategy encompassing terms for both CR and SC, only one retained paper directly measured CR; thus, making the research findings more relevant to SC. Future research could seek to focus on CR to allow for a comparison of findings. Thirdly, the review was conducted early in the COVID-19 pandemic and so does not include more recent publications focusing on resilience specifically in the context of COVID-19. Regardless of this fact, the synthesis of, and recommendations drawn from, the reviewed studies are agnostic to time and specific incident and contain critical elements necessary to address as the pandemic moves from response to recovery. Further research should review the effectiveness of specific interventions during the COVID-19 pandemic for collation in a subsequent update to this current paper. Fourthly, the current review synthesises findings from countries with individualistic and collectivistic cultures, which may account for some variation in the findings. Lastly, despite choosing a scoping review method for ease of synthesising a wide literature base for use by public health emergency researchers in a relatively tight timeframe, there are disadvantages of a scoping review approach to consider: (1) quality appraisal of retained studies was not carried out; (2) due to the broad nature of a scoping review, more refined and targeted reviews of literature (e.g., systematic reviews) may be able to provide more detailed research outcomes. Therefore, future research should seek to use alternative methods (e.g., empirical research, systematic reviews of literature) to add to the evidence base on CR and SC impact and use in public health practice.

## Conclusion

This review sought to establish: (1) How CR and SC are quantified in research?; (2) The impact of community resilience on mental wellbeing?; (3) The impact of infectious disease outbreaks, disasters and emergencies on community resilience and social capital?; and, (4) What types of interventions enhance community resilience and social capital?. The chosen search strategy yielded 26 relevant papers from which we were able extract information relating to the aims of this review.

Results from the review revealed that CR and SC are not measured consistently across research. The impact of CR / SC on mental health and wellbeing during emergencies and disasters is mixed (with some potential for backlash), however the literature does identify cognitive SC as particularly protective. Although only a small number of papers compared CR or SC before and after a disaster, the findings were relatively consistent: SC or CR is negatively impacted by a disaster. Methods suggested to bolster SC in communities were centred around social activities, such as recreational group activities and volunteering. Recommendations for both research and practice (with a particular focus on the ongoing COVID-19 pandemic) are also presented.

### Supplementary Information

**Additional file 1.**

**Additional file 2.**

**Additional file 3.**

**Additional file 4.**

**Additional file 5.**

## Data Availability

The datasets used and/or analysed during the current study are available from the corresponding author on reasonable request.

## Abbreviations

CR: Community resilience
SC: Social Capital
