# Supplementary material for: Examining the role of community resilience and social capital on mental health in public health emergency and disaster response: a scoping review
Source: BMC Public Health. 2023 Dec 12;23:2482. doi: 10.1186/s12889-023-17242-x (PMC10714503; doi:10.1186/s12889-023-17242-x)
Supplement: Supplementary file 1 — Additional file 1. [file 12889_2023_17242_MOESM1_ESM.docx]

**Supplementary File 1**

**Complete search strategy**

**Medline**

Database: Ovid MEDLINE (R) ALL <1946 to 18 May 2020>

Results: 990

|  | **Search Strategy** |
| --- | --- |
| 1 | (Social adj2 (network* or support or capital)).ti,ab. or (community adj2 (capacity or resources)).ab,ti. or connectedness.ab,ti.  (healthy adj2 (communit* or cit*)).ab,ti. or "active citizenship".ab,ti. or ((community or collective) adj2 control).ab,ti. or "empowerment".ab,ti. or (Community adj2 (resilience or asset or strengths or capabilities)).ab,ti. or (Community adaptation adj2 adversity).ab,ti. or ((Resilient or strong) adj2 communit*).ab,ti.or Social resilience.ab,ti.or Health assets.ab,ti.  (Bounce adj2 (back or forward)).ab,ti.or Disaster recovery.ab,ti. or Hardiness.ab,ti. or Neighbourhood resilience.ab,ti. or Mutual aid.ab,ti. or Mutualism.ab,ti. or disaster resilience.ab,ti. |
| 2 | "CBRN*".ab,ti. or Disaster.ab,ti. or "flood*".ab,ti. or volcano.ab,ti. or hurricane.ab,ti. or earthquake.ab,ti. or (Disease* adj3 outbreak*).ab,ti. or "Epidemic*".ab,ti. or "Pandemic*".ab,ti. or (Emergenc* adj3 respon*).ab,ti. or Coronavirus.ab,ti.  COVID-19.ab,ti. or 2019-nCoV.ab,ti. or SARS.ab,ti. or Severe acute respiratory syndrome.ab,ti. or H1N1.ab,ti. or MERS.ab,ti. or MERS-CoV.ab,ti. or Middle East respiratory syndrome.ab,ti. or Ebola.ab,ti. or Swine flu.ab,ti. or Swine influenza.ab,ti. or Avian influenza.ab,ti. or Avian flu.ab,ti. or H7N9.ab,ti. or H1N5.ab,ti. or Grenfell.ab,ti. or (anthrax).ti,ab  (avalanche*).ti,ab or (blizzard*).ti,ab or (chernobyl).ti,ab or (cyclone*).ti,ab or (drought*).ti,ab or (explosion*).ti,ab or  (fire*).ti,ab or (Fukushima).ti,ab or (hurricane*).ti,ab or (industrial accident*).ti,ab or (landslide*).ti,ab or (massacre*).ti,ab  (mass killing).ti,ab or (september 11th).ti,ab or (shooting*).ti,ab or (storm*).ti,ab or (three mile island).ti,ab or (tidal wave*).ti,ab or (tornado*).ti,ab or (tsunami*).ti,ab or (typhoon*).ti,ab or (world trade center).ti,ab |
| 3 | Post traumatic growth.ab,ti. or Post-traumatic growth.ab,ti. or Resilience.ab,ti. or Disaster recovery.ab,ti. or Disaster relief.ab,ti. or Mental health.ab,ti. or Anxiety.ab,ti.or (Wellbeing or well-being).ab,ti. or Coping.ab,ti. |
| 4 | 1 + 2 + 3 |

**EMBASE**

Database: Embase <1974 to 18 May 2020>

Results: 1369

|  | **Search Strategy** |
| --- | --- |
| 1 | (Social adj2 (network* or support or capital)).ti,ab. or (community adj2 (capacity or resources)).ab,ti. or connectedness.ab,ti.  (healthy adj2 (communit* or cit*)).ab,ti. or "active citizenship".ab,ti. or ((community or collective) adj2 control).ab,ti. or "empowerment".ab,ti. or (Community adj2 (resilience or asset or strengths or capabilities)).ab,ti. or (Community adaptation adj2 adversity).ab,ti. or ((Resilient or strong) adj2 communit*).ab,ti.or Social resilience.ab,ti.or Health assets.ab,ti.  (Bounce adj2 (back or forward)).ab,ti.or Disaster recovery.ab,ti. or Hardiness.ab,ti. or Neighbourhood resilience.ab,ti. or Mutual aid.ab,ti. or Mutualism.ab,ti. or disaster resilience.ab,ti. |
| 2 | "CBRN*".ab,ti. or Disaster.ab,ti. or "flood*".ab,ti. or volcano.ab,ti. or hurricane.ab,ti. or earthquake.ab,ti. or (Disease* adj3 outbreak*).ab,ti. or "Epidemic*".ab,ti. or "Pandemic*".ab,ti. or (Emergenc* adj3 respon*).ab,ti. or Coronavirus.ab,ti.  COVID-19.ab,ti. or 2019-nCoV.ab,ti. or SARS.ab,ti. or Severe acute respiratory syndrome.ab,ti. or H1N1.ab,ti. or MERS.ab,ti. or MERS-CoV.ab,ti. or Middle East respiratory syndrome.ab,ti. or Ebola.ab,ti. or Swine flu.ab,ti. or Swine influenza.ab,ti. or Avian influenza.ab,ti. or Avian flu.ab,ti. or H7N9.ab,ti. or H1N5.ab,ti. or Grenfell.ab,ti. or (anthrax).ti,ab  (avalanche*).ti,ab or (blizzard*).ti,ab or (chernobyl).ti,ab or (cyclone*).ti,ab or (drought*).ti,ab or (explosion*).ti,ab or  (fire*).ti,ab or (Fukushima).ti,ab or (hurricane*).ti,ab or (industrial accident*).ti,ab or (landslide*).ti,ab or (massacre*).ti,ab  (mass killing).ti,ab or (september 11th).ti,ab or (shooting*).ti,ab or (storm*).ti,ab or (three mile island).ti,ab or (tidal wave*).ti,ab or (tornado*).ti,ab or (tsunami*).ti,ab or (typhoon*).ti,ab or (world trade center).ti,ab |
| 3 | Post traumatic growth.ab,ti. or Post-traumatic growth.ab,ti. or Resilience.ab,ti. or Disaster recovery.ab,ti. or Disaster relief.ab,ti. or Mental health.ab,ti. or Anxiety.ab,ti.or (Wellbeing or well-being).ab,ti. or Coping.ab,ti. |
| 4 | 1 + 2 + 3 |

**PsycInfo**

Database: Embase <1806 to 18 May 2020>

Results: 1225

|  | **Search Strategy** |
| --- | --- |
| 1 | (Social adj2 (network* or support or capital)).ti,ab. or (community adj2 (capacity or resources)).ab,ti. or connectedness.ab,ti.  (healthy adj2 (communit* or cit*)).ab,ti. or "active citizenship".ab,ti. or ((community or collective) adj2 control).ab,ti. or "empowerment".ab,ti. or (Community adj2 (resilience or asset or strengths or capabilities)).ab,ti. or (Community adaptation adj2 adversity).ab,ti. or ((Resilient or strong) adj2 communit*).ab,ti.or Social resilience.ab,ti.or Health assets.ab,ti.  (Bounce adj2 (back or forward)).ab,ti.or Disaster recovery.ab,ti. or Hardiness.ab,ti. or Neighbourhood resilience.ab,ti. or Mutual aid.ab,ti. or Mutualism.ab,ti. or disaster resilience.ab,ti. |
| 2 | "CBRN*".ab,ti. or Disaster.ab,ti. or "flood*".ab,ti. or volcano.ab,ti. or hurricane.ab,ti. or earthquake.ab,ti. or (Disease* adj3 outbreak*).ab,ti. or "Epidemic*".ab,ti. or "Pandemic*".ab,ti. or (Emergenc* adj3 respon*).ab,ti. or Coronavirus.ab,ti.  COVID-19.ab,ti. or 2019-nCoV.ab,ti. or SARS.ab,ti. or Severe acute respiratory syndrome.ab,ti. or H1N1.ab,ti. or MERS.ab,ti. or MERS-CoV.ab,ti. or Middle East respiratory syndrome.ab,ti. or Ebola.ab,ti. or Swine flu.ab,ti. or Swine influenza.ab,ti. or Avian influenza.ab,ti. or Avian flu.ab,ti. or H7N9.ab,ti. or H1N5.ab,ti. or Grenfell.ab,ti. or (anthrax).ti,ab  (avalanche*).ti,ab or (blizzard*).ti,ab or (chernobyl).ti,ab or (cyclone*).ti,ab or (drought*).ti,ab or (explosion*).ti,ab or  (fire*).ti,ab or (Fukushima).ti,ab or (hurricane*).ti,ab or (industrial accident*).ti,ab or (landslide*).ti,ab or (massacre*).ti,ab  (mass killing).ti,ab or (september 11th).ti,ab or (shooting*).ti,ab or (storm*).ti,ab or (three mile island).ti,ab or (tidal wave*).ti,ab or (tornado*).ti,ab or (tsunami*).ti,ab or (typhoon*).ti,ab or (world trade center).ti,ab |
| 3 | Post traumatic growth.ab,ti. or Post-traumatic growth.ab,ti. or Resilience.ab,ti. or Disaster recovery.ab,ti. or Disaster relief.ab,ti. or Mental health.ab,ti. or Anxiety.ab,ti.or (Wellbeing or well-being).ab,ti. or Coping.ab,ti. |
| 4 | 1 + 2 + 3 |
