# Supplementary material for: Examining the role of community resilience and social capital on mental health in public health emergency and disaster response: a scoping review
Source: BMC Public Health. 2023 Dec 12;23:2482. doi: 10.1186/s12889-023-17242-x (PMC10714503; doi:10.1186/s12889-023-17242-x)
Supplement: Supplementary file 2 — Additional file 2. [file 12889_2023_17242_MOESM2_ESM.docx]

**Supplementary File 2**

**Final inclusion and exclusion criteria used for record screening**

| Include | Exclude |
| --- | --- |
| Include articles that use general public / population samples. | Exclude articles which use a healthcare or specialist population sample, or where data is inseparable for general population and specialist. |
| Include articles relating to public health emergencies or infectious disease outbreaks which affect communities (on either a local or national level). | Exclude articles relating to public health emergencies or infectious disease outbreaks which affect individual cases. |
| Include articles which assess impact of community resilience or social capital on mental health and wellbeing, resilience, and recovery during and following public health emergencies and infectious disease outbreaks. | Exclude studies that simply refer to measurement of perceived social support alone within Title/Abstract without explicit reference to communities. |
| Include articles that use primary research. | Exclusion of studies which focus on workplace settings (including schools). |
| Include articles which have the full text accessible. | Exclusion of articles which focus on preparedness. |
|  | Exclusion of non-peer reviewed articles. |
|  | Exclude review articles. |
|  | Exclusion of all non-English language articles. |
