# Supplementary material for: Examining the role of community resilience and social capital on mental health in public health emergency and disaster response: a scoping review
Source: BMC Public Health. 2023 Dec 12;23:2482. doi: 10.1186/s12889-023-17242-x (PMC10714503; doi:10.1186/s12889-023-17242-x)
Supplement: Supplementary file 3 — Additional file 3. [file 12889_2023_17242_MOESM3_ESM.docx]

**Supplementary File 3**

**Eligibility criteria, screening process and data charting**

| **Eligibility criteria** |
| --- |
| **Inclusion criteria** |
| - Include articles that use general public / population samples. - Include articles relating to public health emergencies or infectious disease outbreaks which affect communities (on either a local or national level). - Include articles which assess impact of community resilience or social capital on mental health and wellbeing, resilience, and recovery during and following public health emergencies and infectious disease outbreaks. - Include articles that use primary research. - Include articles which have the full text accessible. |
| **Exclusion criteria** |
| - Exclusion of non-peer reviewed articles. - Exclusion of articles which focus on preparedness. - Exclusion of studies which focus on workplace settings (including schools). - Exclude studies that simply refer to measurement of perceived social support alone within Title/Abstract without explicit reference to communities. - Exclude articles relating to public health emergencies or infectious disease outbreaks which affect individual cases. - Exclude articles which use a healthcare or specialist population sample, or where data is inseparable for general population and specialist. - Exclude review articles. - Exclusion of all non-English language articles. |

**Title/abstract screening**

- Yes OR maybe meets eligibility criteria (1 reviewer) 🡪 Full text screening.
- No does not meet eligibility criteria (1 reviewer) 🡪 exclude.

**Full text screening for inclusion and exclusion**

- Yes meets eligibility criteria (1 reviewer) 🡪 Full text screening.
- Maybe meets eligibility criteria (1 reviewer) 🡪 reviewed by second reviewer.
- No does not meet eligibility criteria (1 reviewer) 🡪 exclude.

**Data charting**

| **Title** | **Authors** | **Origin** | **Year** | **Study design** | **Aim** | **Disaster** | **N** | **Characteristics** | **Variables examined** | **Results** | **Restrictions / Limitations** | **Recommendations** |
| --- | --- | --- | --- | --- | --- | --- | --- | --- | --- | --- | --- | --- |
|  |  |  |  |  |  |  |  |  |  |  |  |  |
