# Supplementary material for: Examining the role of community resilience and social capital on mental health in public health emergency and disaster response: a scoping review
Source: BMC Public Health. 2023 Dec 12;23:2482. doi: 10.1186/s12889-023-17242-x (PMC10714503; doi:10.1186/s12889-023-17242-x)
Supplement: Supplementary file 4 — Additional file 4. [file 12889_2023_17242_MOESM4_ESM.docx]

**Supplementary File 4**

**Data extracted from papers which reported a negative effect of community resilience or social capital on mental health or wellbeing.**

| **Reference** | **Finding** | **Suggested explanation from paper** |
| --- | --- | --- |
| (1) | - CR showed a positive association with anxiety and mood symptom treatments. | - There may be individuals that do not benefit from strong development or connectedness - Christchurch community was both resilient and vulnerable at the same time, e.g. increased stress but increased community activities too. - In Christchurch communities despite the great work of community organisations, there has been a lot of uncertainty and distress caused by the severe disruptions to the built and economic environments, as well as a loss of trust in the recovery and political decision-making as a result of poor communication, inappropriate support and lack of community involvement indecision-making |
| (2) | - At the community-level, living in an area with higher SC was significantly associated with higher posttraumatic stress. | - Although we had conceptualized a lower percentage of residents living alone as an indicator of higher SC, it might have been an inadequate proxy for the construct. It could be, for example, that a relatively higher percentage of residents living alone fosters greater connections between residents, thus leader to higher SC. |
| (3) | - high levels of social participation at community-level were positively associated with the risk of depression among women. | - Pressure of expectations to help and support others. - Being isolated by the in-group. - While women are more likely to be protectively affected by social cohesion at the community level. Given that women tend to have larger and more diverse social networks than men. |
| (4) | - immediately after the storm, the link between SC and stress is positive (more SC means more stress), then eventually turns more negative (more SC means less stress) | - Those with greater SC have greater social networks and social support during ordinary times that enable them to handle stress better. During the height of a crisis, this same social embeddedness places extraordinary demands and burdens on them, as they try to help and support their wide networks of family, friends, neighbours, colleagues, and community members. As the recovery from the crisis proceeds, those with greater SC are able to snap back more effectively and handle stress better, as they begin to routinize their activities and once again rely more on their social networks for support. |
| (5) | - participation in social structures (i.e. structural SC) may be associated with an excess of anxiety disorders | - Whereas depressed individuals symptomatically avoid structural involvement in social networks, anxious people may seek reassurance for their anxious feelings and thoughts. As a result, those individuals who show the greatest anxiety may have a larger network (i.e. higher structural SC) to address their needs. - In a disaster situation intimate social involvement within one’s network may predispose individuals to the ‘contagion of stress’ when stressful life events afflict those whom they feel emotionally close. This may lead to increased feelings of anxiety. |

**References**

1. Hogg D, Kingham S, Wilson TM, Ardagh M. The effects of spatially varying earthquake impacts on mood and anxiety symptom treatments among long-term Christchurch residents following the 2010/11 Canterbury earthquakes, New Zealand. Health Place. 2016;41:78-88.

2. Lowe SR, Sampson L, Gruebner O, Galea S. Psychological resilience after Hurricane Sandy: the influence of individual- and community-level factors on mental health after a large-scale natural disaster. PLoS ONE. 2015;10(5):e0125761.

3. Sato K, Amemiya A, Haseda M, Takagi D, Kanamori M, Kondo K, et al. Post-disaster Changes in Social Capital and Mental Health: A Natural Experiment from the 2016 Kumamoto Earthquake. American journal of epidemiology. 2020;30.

4. Weil F, Lee MR, Shihadeh ES. The burdens of social capital: How socially-involved people dealt with stress after Hurricane Katrina. Social Science Research. 2012;41(1):110-9.

5. Wind T, Fordham M, Komproe IH. Social capital and post-disaster mental health. Glob Health Action. 2011;4.
