# Supplementary material for: Examining the role of community resilience and social capital on mental health in public health emergency and disaster response: a scoping review
Source: BMC Public Health. 2023 Dec 12;23:2482. doi: 10.1186/s12889-023-17242-x (PMC10714503; doi:10.1186/s12889-023-17242-x)
Supplement: Supplementary file 5 — Additional file 5. [file 12889_2023_17242_MOESM5_ESM.docx]

**Supplementary file 5**

**Retailed papers methods of measuring mental wellbeing**

| **Authors** | **Outcome variables** |
| --- | --- |
| (59) | Self-efficacy; *We operationalise self-efficacy as a conjoint measure of two items, both capturing a household’s perceived ability to protect against flooding*. |
| (60) | Post Traumatic Stress symptoms; *PTSD Checklist for DSM-V (PCL-5; Weathers et al., 2013) a self-report measure* Depression symptoms; *The Center for Epidemiologic Studies Depression Scale Revised (CESD-R;Eaton, Smith,Ybarra,Muntaner, & Tien, 2004).* Life satisfaction; *the Satisfaction with Life Scale (SWLS; Diener, Emmons, Larsen, & Griffin, 1985)* |
| (57) | PTSD-related symptoms using a previously validated Spanish version of the PCL-C (Miles, Marshall, & Schell, 2008; Vera-Villarroel, Zych, Celis-Atenas, Córdova-Rubio, & Buela-Casal, 2011). |
| (35) | Post Traumatic Stress symptoms; *Post-traumatic Stress Disorder Symptom Scale-Self Report (PSS-SR) was utilized to assess current symptoms of posttraumatic stress resulting from Hurricane Sandy (Foa, Riggs, Dancu, & Rothbaum, 1993)* Depressive symptoms; *the 10-item short form of the Center for Epidemiologic Studies Depression Scale (CES-D; Andresen, Malmgren, Carter, & Patrick, 1994).* |
| (42) | Cognitive decline; *standardized in-home assessment under the Japanese Long-Term Care Insurance (LTCI) scheme established in 2000 (Tamiya et al., 2011).* |
| (36) | PTSD symptomatology; *assessed using the Screening Questionnaire for Disaster Related Mental Health* |
| (56) | Mood and anxiety; *Measures of mood and anxiety consisted of treatment information among care-seekers enroled in a PHO. It needs to be kept in mind that this measure includes not only treatment for diagnosed mood and anxiety disorders but also treatment ofmood and anxiety symptomatology, which is mostly done in the case of moderate to severe symptoms. For simplicity, we just refer to “mood and anxiety symptom treatment”. [...]* ***These individual level indicators were then further aggregated to the community level to get yearly counts of mood and anxiety symptom treatments per community.*** |
| (53) | Life satisfaction; *The Satisfaction with Life Scale (SWLS;Diener, Emmons, Larsen, & Griffin, 1985) is a five-item instrument that measures life satisfaction. In this study, we used the Chinese version ofthe SWLS, as translated by Shek (1998).* Depression; *The Center for Epidemiologic Studies Depression Scale (CES-D; Radloff, 1977) is a 20-item scale designed to measure depression. In this study, we used the 10-item Chinese version of the CES-D, as translated by Wong (2009).* |
| (43) | Subjective wellbeing; *measured by the Personal Wellbeing Index (PWI).* |
| (38) | Posttraumatic Growth; *measured using the self-rated measure of the short form of the Posttraumatic Growth Inventory (PTGI-SF; Cann et al., 2010)* Psychological Resilience; *The two-item Connor-Davidson Resilience Scale (CD-RISC-2; Vaishnavi, Connor, & Davidson, 2007) was used to measure levels of psychological resilience.*  Loneliness; *The eight-item UCLA Loneliness scale (ULS-8; Hays & DiMatteo, 1987) was used to measure subjective feelings of loneliness and social isolation.* Depression; *The Center for Epidemiologic Studies—Depression scale (CES–D; Radloff, 1977) was used to measure respondents’ levels of depressive symptoms.* |
| (37) | Psychological Resilience; *The 10-item Connor-Davidson Resilience Scale (CD-RISC 10)39 was used to measure levels of psychological resilience.* Depressive Symptoms; *The Center for Epidemiologic Studies Depression scale40 was used to measure depressive symptoms.* |
| (39) | Disaster-related posttraumatic stress; *assessed using the PTSD Checklist for DSM-5 (PCL-5), a 20-item inventory measuring symptoms ofPTSD as defined in the Diagnostic and Statistical Manual ofMental Disorders, Fifth Edition (DSM-5)* Depression; *assessed using the nine-item Patient Health Questionnaire (PHQ-9)* |
| (47) | Psychological distress; K6 scale developed by Kessler et al. (Kessler et al., 2002, 2003) |
| (48) | Mood/anxiety disorder; *assessed through the self-administered Japanese version of the K6.* |
| (58) | Symptoms of Post-Traumatic Stress Disorder; *assessed by the National Stressful Events Survey PTSD Short Scale (APA, 2013).* |
| (40) | Depression; *assessed with the validated 20item Center for Epidemiological Studies Depression (CESD) Scale (Radloff, 1977).* |
| (49) | PTSD Symptoms and Major depressive episode; *assessed using the Screening Questionnaire for Disaster Mental Health (SQD)* |
| (44) | Happiness; *The 2015 SSLDR used one questionnaire item (“In general, do you feel happy?”) to measure individuals’ happiness.* |
| (50) | Psychological distress; *we assessed psychological distress according to the Kessler 6 (K6) scale.* |
| (41) | Variety of indicators of stress; *e.g.,* *For generalized Emotional stress, we asked if people felt depressed or angry. For stressful feelings regarding Fear of Social Disorder and Evacuees, we asked a question about their potential fear toward evacuees.* […] *also asked a* *series of questions about their feelings of aggravation about traffic congestion, crowding in neighborhoods, stores, and schools, telephone disruptions, and rudeness.* |
| (54) | Anxiety and depression; *Symptoms of anxiety and depression were assessed by the Hopkins Symptom Checklist-25 [HSCL-25 (34)].* Posttraumatic Stress Disorder; *Symptoms of PTSD were assessed by the PTSD Checklist Civilian Version (PCL-C) (35).* |
| (55) | Posttraumatic Stress; *assessed by the PTSD Checklist Civilian Version (PCL-C; Weathers, Litz, Herman, Huska, & Keane, 1993).* |
| (45) | Depression; *measured using the ten-item Center for Epidemiologic Studies Depression Scale (CES-D).* Life satisfaction; *measured using the five-item Satisfaction with Life Scale (SWLS) developed by Diener et al. (1985).* |
| (46) | Anxiety; *SAS questionnaire was used to measure the levels of anxiety of the study participants.* Stress; *SASR questionnaire using a six-point Likert scale, which contained 30 items, was used to measure stress.* |

*Note*. Italic text is quoted directly from the cited research paper.
